# Assessment of refractive errors, amblyopia, strabismus, and low vision among hearing-impaired and deaf students in Kermanshah

**DOI:** 10.1186/s12886-024-03515-5

**Published:** 2024-06-12

**Authors:** Haleh Kangari, Amir Etemadi Majd, Mohammad Ghasemi Broumand, Seyed Mehdi Tabatabaee

**Affiliations:** Shahid Beheshti University of Medical Sciences, Tehran, Iran

**Keywords:** Amblyopia, Hearing-impaired, Kermanshah, Low vision, Refractive errors, Strabismus

## Abstract

**Background and Aim:**

Refractive errors, amblyopia, strabismus, and low vision are more common among children with hearing impairments in comparison with their hearing peers. Neglecting visual disorders can pose educational and social problems for these children. The present study aimed to assess the prevalence of refractive errors, amblyopia, strabismus, and low vision among hearing-impaired and deaf students in Kermanshah.

**Materials and methods:**

A total of 79 deaf and hearing impaired students within the age range of 7–20 years (mean age of 15.01 ± 2.72) underwent optometric examinations, including autorefractometry, retinoscopy, ophthalmoscopy, slit lamp, visual acuity measurement, and cover-uncover test. Those who needed further evaluation were referred to the Ophthalmology Clinic of Imam Khomeini Hospital.

**Results:**

Regarding the prevalence of refractive errors, 32 (40.5%) subjects had one or a combination of refractive errors, the most common of which was astigmatism (36.7%), followed by amblyopia (15.1%). The most common type of strabismus was latent strabismus (heterophoria) (88.6%), followed by exophoria (81%). Moreover, 3 (3.7%) cases had nystagmus. A significant difference was observed between the prevalence of amblyopia and the degree of hearing loss (*P* = 0.026), and no significant difference was detected in other cases.

**Conclusion:**

As evidenced by the obtained results, refractive errors, amblyopia, strabismus, and low vision are more prevalent among deaf and hearing-impaired children compared to normal children because deaf and hearing-impaired children are not able to convey their vision problems and need to compensate for their poor hearing with an enhanced sense of sight, inattention to these disorders can present these children with serious educational and social problems. Therefore, eye screening examinations are of paramount importance in deaf and hearing-impaired children.

## Introduction

Vision and hearing are by far the most important human senses that help us to take in the world around us. When one of these two senses suffers a serious defect, the other sense rises in importance [1]. Defects in the hearing system cause a disturbance in one’s ability to communicate with others, resulting in a failure in communication and social interactions. Hearing impairment is sorted into two major categories: hearing loss and deafness. According to the World Health Organization, a deaf individual is described as a person who is unable to process auditory information with or without the use of hearing aids, and a hearing-impaired person is someone capable of auditory processing with the use of hearing aids [2–5].

As detailed by researchers, there is no single cause for hearing loss; rather, a wide range of issues are at play, including congenital and hereditary diseases, infections, and traumas, affecting different parts of the ear and its mechanisms. It has been observed that 10-15% of children are rejected in screening programs; therefore, it is of utmost importance to recognize the causes of hearing loss, the types of damage, the degrees of these damages, and the subsequent impact on the child’s performance. It is also necessary to be fully aware of other factors associated with hearing loss, such as race, age, gender, and socioeconomic characteristics, and assess their effects on hearing loss in the final assessment.

Hearing loss is a hidden disability in children, especially in neonates and toddlers, and considering that human hearing is complete at 12 months, undetected and untreated hearing loss in this population leads to speech and language delays, as well as emotional, social, and academic problems. Like adults, children experience different degrees of hearing loss, each leading to specific problems for the child [6]. Hearing-impaired and deaf individuals rely on their visual senses more than healthy people; therefore, even low degrees of refractive error, if uncorrected, can reduce visual performance and present people with serious problems [7]. In addition, previous studies that assessed visual disorders among the deaf and hearing impaired demonstrated that the prevalence of refractive errors, amblyopia, strabismus, and low vision among deaf and hearing-impaired children is higher in comparison with their hearing peers [3, 4, 7].

The prevalence of vision problems among normal-hearing people is 17-30%, while it is 44-65% among hard of hearing and deaf people [7, 8]. The higher number of vision disorders in deaf and hearing-impaired people compared to normal-hearing people can be attributed to the fact that the retina and cochlea originate from a single layer in the sixth and seventh weeks of the embryonic period. They may be affected by genetic and environmental factors, such as lack of oxygen, toxic agents, and viruses [1, 4, 5, 7]. Rogers observed that the prevalence of vision problems rises with increased severity of hearing loss. The noteworthy issue was the relatively high prevalence of vision disorders in hearing loss associated with congenital rubella syndrome [8].

The studies conducted on deaf-blind children indicated the following points: (1) A high prevalence of neurological disabilities, including neuromuscular disorders, is observed in congenitally deaf-blind children; (2) Severe hearing impairment is more common than severe visual impairment in deaf-blind children; (3) Many deaf-blind children have normal peripheral hearing without normal behavioral responses to sound; and (4) The combination of auditory and visual conflicts, along with neurological disabilities, have profound effects on language and cognitive abilities, as well as the overall development of the child [6].

As mentioned earlier, deaf and hearing-impaired children rely on the sense of sight to understand the world around them and develop effective communication skills, such as learning sign language and lip reading. Due to the higher prevalence of vision disorders in these children, it is necessary that all of them undergo a comprehensive eye exam immediately after the diagnosis of deafness so that their vision disorders can be diagnosed and resolved. Early diagnosis and correction of vision disorders among deaf and hearing impaired children, the correction of correctable problems, such as eyeglass prescriptions in refractive errors, and treatment of curable problems, such as cataract surgery, increase visual performance exert a positive effect on the process of learning, education, and communication. Binocular vision dysfunction can be prevented and treated if diagnosed in childhood [2, 5, 7].

Although the prevalence of vision disorders in deaf and hearing-impaired students has been proven, the prevalence and types of these disorders are different in various regions across the globe. Despite the importance of early detection of vision disorders in deaf and hearing-impaired students, few studies have been conducted in this regard in our country. Consequently, it is of utmost importance to conduct studies on vision disorders in these students, considering the impact of genetic and environmental factors, as well as people’s lifestyle, on the prevalence and type of these disorders.

In light of the aforementioned issues, the present study aimed to assess the type and prevalence of refractive errors, strabismus, amblyopia, and low vision in deaf and hearing-impaired students in Kermanshah in the academic year 2018–2019.

## Materials and methods

This cross-sectional study was conducted in primary and secondary schools for deaf and hearing-impaired students in Kermanshah in the 2018–2019 academic year. The ethics code of this research project is IR.SBMU.RETECH.REC.1398.822. The data were analyzed using SPSS statistical software (version 20). The variables were described as mean and standard deviation. Moreover, two-way analysis of variance (ANOVA) was employed to analyze the data. To test the hypotheses, the alpha, or significance level, was set to 0.05 (5%), and the student’s t-test was used to compare the mean in the studied sample.

The research was initiated after necessary coordination with the School of Rehabilitation, Shahid Beheshti University of Medical Sciences, the Education Department of Kermanshah, and schools for deaf and hard-of-hearing students. Every day, 1–5 deaf, hearing impaired, and normal-hearing.

students were sent from the schools to the optometry office for examination. A total of 39 deaf and hearing impaired students within the age range of 7–20 years (mean age: 15.01 ± 2.72) (Table 1) underwent a thorough optometric examination, including autorefractometry, retinoscopy, ophthalmoscopy, slit lamp, and visual acuity measurement, which can determine, to some extent, the type and severity of refractive errors.

The assessment of visual acuity by the pinhole test makes it possible to differentiate between reduced visual acuity caused by pathological diseases and refractive errors. If the pinhole does not improve visual acuity, the decreased vision is probably due to the presence of pathological eye diseases. The Snellen chart was used to assess children’s visual acuity, and less than 20/30 vision was considered reduced vision. Retinoscopy is the most effective objective technique for evaluating refractive status, especially in children and non-cooperative patients.

Accommodation increases the refractive power of the eye up to 15 diopters. Paralysis of accommodation (by cycloplegic drugs) can help the assessment of refractive errors [9]. The cover test was used to determine the type of strabismus, and the amount of deviation was measured by the alternate prism cover test and the Krimsky method. A deviation greater than 10 prism diopters was considered strabismus. If there was a refractive error, eyeglasses were prescribed for the children. Hyperopia was greater than or equal to + 1.00 diopters, myopia was greater than or equal to -0.5 diopters, and astigmatism was greater than or equal to -0.75 diopters. Anisometropia was considered a difference of 1 or more diopters in the amount of refractive error between the two eyes.

Amblyopia was regarded as vision less than 20/30 with the best optical correction or an interocular difference of 2 lines or more in a visual acuity table. A visual acuity of 20/200 was considered severe low vision [10]. The examination results were registered in a form prepared in advance. For detailed eye examinations, students with vision problems were referred to the Ophthalmology Clinic of Imam Khomeini Hospital in Kermanshah. In order to increase the accuracy of the research, the same steps were carried out in a regular school where elementary and middle school students were studying.

## Results

Regarding the prevalence of refractive errors, 32 (40.5%) subjects had one or a combination of refractive errors. The most common refractive error among them was astigmatism (36.7%) (Table 3), followed by amblyopia (15.1%) (Table 5). The most common type of strabismus was latent strabismus (heterophoria) (88.6%), followed by exophoria (81%). Moreover, 3 (3.7%) cases had nystagmus (Table 4). Furthermore, 3 (3.7%), 70 (88.6%), and 9 (11.3%) subjects had low vision, severe hearing loss, and moderate hearing loss (Table 2), respectively. A significant difference was observed between the prevalence of amblyopia and the degree of hearing loss (*P* = 0.026) (Table 5), and no significant difference was observed in other cases. A number of 84 male students studying in a regular school were examined as the control group. Their mean age was 11 years, and 15 (17.8%) of them had refractive errors.

**Table 1:** Distribution of mean and standard deviation of age, visual acuity, and refractive errors of deaf and hearing-impaired students in Kermanshah in the academic year 2019-2020

	Mean	standard deviation	Range	Minimum	Maximum
Age	15.1	2.72	13	7	20
Right eye visual acuity	0.80	0.30	0.96	0.04	1
Left eye visual acuity	0.82	0.29	0.98	0.02	1
Hyperopia	0.18	0.57	4	0	4
Myopia	0.43	1.1	6	0	6
Astigmatism	0.66	1.06	4.5	0	4.5

Mean and standard deviation of age: 15.01 ± 2.72

Mean and standard deviation of hyperopia: 0.18 ± 0.57

Mean and standard deviation of right eye visual acuity: 0.80 ± 0.30

Mean and standard deviation of myopia: 0.43 ± 1.1

Mean and standard deviation of left eye visual acuity: 0.82 ± 0.29

Mean and standard deviation of astigmatism: 0.66 ± 1.06

**Table 2:** Distribution of degree of hearing loss by gender in deaf and hearing impaired students of Kermanshah in the academic year 2019-2020

Degree of hearing loss	Gender		Total
	Male	Female	
Sever	43	27	70
Moderate	6	3	9
Total	49	30	79

The p-value was obtained at 0.761, and no significant difference was observed between the degree of hearing loss and gender

**Table 3:** Incidence of astigmatism in deaf and hearing-impaired students in Kermanshah in the academic year 2019–2020

Degree of hearing loss	Astigmatism		Total
	No	Yes	
Severe	47	23	70
Moderate	3	6	9
Total	50	29	79

The p-value was obtained at 0.068, and no significant difference was observed between the degree of hearing loss and astigmatism

**Table 4:** Incidence of strabismus in deaf and hard of hearing students in Kermanshah in the academic year 2019-2020

Degree of hearing loss	Strabismus				Total
	Orthophoria	Exophoria	Esophoria	Nystagmus	
Severe	5	59	4	2	70
Moderate	1	5	2	1	9
Total	6	64	6	3	79

The p-value was obtained at 0.131, and no significant difference was observed between the degree of hearing loss and strabismus

**Table 5:** Prevalence of amblyopia in deaf and hard of hearing students in Kermanshah in the academic year 2019-2020

Degree of hearing loss	Amblyopia		Total
	No	Yes	
Severe	62	8	70
Moderate	5	4	9
Total	67	12	79

The p-value was obtained at 0.026, and significant difference was observed between the degree of hearing loss and amblyopia

## Discussion

The prevalence of refractive errors, amblyopia, strabismus, and low vision is more common among deaf and hearing-impaired children than their hearing peers. In previously conducted studies, the prevalence of these problems among deaf and hearing-impaired children has been reported as 44–65%. In the present study, among 79 deaf and hearing-impaired primary and secondary school students who were examined for vision, 32 (40.5%) cases had one or a combination of refractive errors, the most common of which was astigmatism (36.7%). Moreover, three of them had albinism, nystagmus, and low vision. In terms of strabismus, tropia was not detected in them, and all of them had phoria.

A notable issue observed in this research was a failure to give close attention to the vision disorders of these children due to various reasons, such as parental neglect, students’ inattention, and their family’s financial problems, resulting in their academic and communication failure. A comparison between Kermanshah and larger cities, such as Tehran, Mashhad, and Isfahan, in terms of the prevalence of refractive errors, amblyopia, strabismus, and low vision in deaf and hearing-impaired children gives us an insight into the high prevalence of these problems in the aforementioned population in Kermanshah. This finding can be ascribed to more consanguineous marriages and subsequent genetic problems in this city.

Moreover, their unfavorable living environment and maternal poor nutritional status during pregnancy are influential in increasing the prevalence of these problems. According to the observations in this research, most of these children belonged to families with low income levels; therefore, they may not have received healthy food for growth during pregnancy since, according to researchers, genetics and environment are responsible for the increased risk of disease outbreaks in a region. For instance, we compare the prevalence of refractive errors and amblyopia among deaf and hearing-impaired children in Tehran and Kermanshah.

In a study conducted by Khorrami-Nejad et al. in 2017 in Tehran on 158 deaf students, the prevalence of refractive errors was reported as 39.9%. In the present research, the prevalence of refractive errors was 40.5%, indicating the higher prevalence of refractive errors in deaf and hard of hearing children in Kermanshah compared to that in Tehran. The prevalence rates of amblyopia were reported as 13.9% and 15.1% in the study by Khorrami-Nejad et al. and the current research, suggesting the higher prevalence of amblyopia is in deaf and hard of hearing children in Kermanshah in comparison with that in Tehran.

As a result, more genetic and environmental problems in Kermanshah may have been responsible for this increase. The results obtained in this research were consistent with the findings of previous studies [11]. In a study by Mohammadi in 2013 on deaf and hearing-impaired students in Sanandaj, the prevalence of refractive errors was reported as 39.5% [12]. In another study conducted in Turkey by Hanioglu-Kargi et al. in 2002 on deaf and hard of hearing students, refractive errors were reported as 29.8%, and astigmatism was more common than other refractive errors [5].

In another study by Bakhshaee et al. in Mashhad in 2002, the prevalence of refractive errors was reported as 28% [13]. In a study carried out by Ostadi Moghadam et al. (2015) in Mashhad on 254 hearing-impaired people, the prevalence of amblyopia was reported as 12.2% [14]. In another study conducted in Turkey by Hanioglu-kargi et al. in 2002, the prevalence of amblyopia was reported as 15.3% [5]. In another study by Abtahi et al. in 2016–2017, the prevalence of amblyopia was reported as 7.8% [15]. The prevalence of low vision in this study was 3.8%. In the study by Mohammadi in Sanandaj, the prevalence of low vision was reported as 2.41% [11]. In another study conducted in Ghana in 2011 by Ovenseri-Ogbomo et al. on deaf and hearing-impaired students, the prevalence of visual impairment was reported as 5.7% [16].

## Conclusion

As evidenced by the obtained results, refractive errors, amblyopia, strabismus, and low vision are more prevalent among deaf and hearing-impaired children compared to their normal-hearing peers. Since deaf and hearing-impaired children have difficulty conveying their vision problems and need to compensate for their poor hearing with an enhanced sense of sight, inattention to these disorders poses serious educational and social problems for these children; therefore, eye screening examinations are paramount in these children.

It is recommended that a comparative analysis be conducted among other areas of children in future studies.

## Data Availability

data available from the corresponding author upon reasonable request.
